# The Phenotypic and Genotypic Spectrum of *BRPF1*‐Related Disorder: 29 New Patients and Literature Review

**DOI:** 10.1111/cge.14688

**Published:** 2024-12-29

**Authors:** Cindy Colson, Marine Tessarech, Elise Boucher‐Brischoux, Odile Boute‐Benejean, Catherine Vincent‐Delorme, Clémence Vanlerberghe, Simon Boussion, Justine Le Cunff, Bénédicte Duban‐Bedu, Laurence Faivre, Christel Thauvin, Christophe Philippe, Ange‐Line Bruel, Frédéric Tran Mau‐Them, Clara Houdayer, Gaetan Lesca, Audrey Putoux, Jonathan Lévy, Olivier Patat, Marlène Rio, Jamal Ghoumid, Thomas Smol

**Affiliations:** ^1^ Univ. Lille, CHU Lille, ULR7364 – RADEME – Maladies RAres du DEveloppement embryonnaire et du Métabolisme CRMR Déficiences Intellectuelles de Causes Rares Lille France; ^2^ CHU Angers Service de Génétique Médicale Angers France; ^3^ CHU Besançon Centre de Génétique Humaine Besançon France; ^4^ Hôpital Saint Vincent de Paul, GHICL Centre de Génétique Chromosomique Lille France; ^5^ UMR1231 GAD, Inserm, Université Bourgogne‐Franche Comté Dijon France; ^6^ CRMR Anomalies du Développement et Syndromes Malformatifs Centre de Génétique, FHU‐TRANSLAD, CHU Dijon Bourgogne Dijon France; ^7^ CHU Dijon Bourgogne, Service de Génomique Médicale Laboratoire de Biologie Médicale Dijon France; ^8^ Hospices Civils de Lyon, Service de Génétique Université Claude Bernard Lyon 1 Lyon France; ^9^ Département de Génétique Hôpital Universitaire Robert‐Debré Paris France; ^10^ Département de Génétique médicale CHU Toulouse Toulouse France; ^11^ Service de génétique Hôpital Necker Paris France

**Keywords:** BRPF1, IDDDFP, neurodevelopmental disorders, phenotypic variability

## Abstract

Intellectual Developmental Disorder with Dysmorphic Facies and Ptosis (IDDDFP) is a rare autosomal dominant syndrome caused by pathogenic variants in the *BRPF1* gene, which is critical for chromatin regulation. This study expands the clinical and molecular spectrum of IDDDFP by analysing 29 new patients from 20 families with confirmed *BRPF1* variants. Our cohort presented with a wide range of clinical features including developmental delay, intellectual disability (ID) and characteristic dysmorphic facial features such as ptosis, blepharophimosis and a broad nasal bridge. New phenotypic features identified include palpebral oedema, laterally elongated eyebrows, low hanging columella and hypertrichosis. Neuropsychological assessment reveals a predominance of mild to moderate ID, with cognitive profiles showing variability in verbal and visual processing. Structural abnormalities such as agenesis of the corpus callosum and ocular defects were noted, consistent with previous studies but with some differences. Familial analysis revealed variability in clinical expression. Our findings highlight the diverse clinical manifestations of *BRPF1*‐related disorders and suggest that comprehensive ophthalmological evaluation is essential for the management of these patients.

## Graphical abstract

This study analyses 29 new Intellectual Developmental Disorder with Dysmorphic Facies and Ptosis (IDDDFP) patients with *BRPF1* variants and reveals a broad phenotypic spectrum, including novel features such as palpebral oedema. It highlights the variability in clinical expression, underlines the importance of ophthalmological assessment and detailed familial neuropsychological profiling for improved patient management.

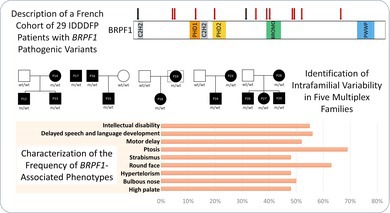

## 1 Introduction

Intellectual disability (ID) is a common neurodevelopmental disorder (NDD) and is a frequent indication for genetic counselling. ID can present either as a non‐syndromic condition or as part of a syndromic condition with additional clinical features. Advances in genomic technologies have led to the identification of about 2000 genes that are either implicated in, or candidates for, different forms of ID [1, 2, 3]. Untargeted approaches such as exome sequencing (ES) or genome sequencing (GS) have shown that pathogenic variants in a single gene can be associated with a wide range of neurobehavioral, neurodevelopmental and physical phenotypes.

Heterozygous pathogenic variants resulting in haploinsufficiency of the chromatin regulator gene *BRPF1* (bromodomain‐ and plant homeodomain‐linked zinc finger‐containing protein 1; MIM #602410) have been identified as the cause of Intellectual Developmental Disorder with Dysmorphic Facies and Ptosis (IDDDFP; MIM #617333) [4, 5]. IDDDFP is a rare autosomal dominant syndrome characterised by developmental delay, intellectual disability, speech and language impairment, ophthalmological abnormalities and dysmorphic facial features such as ptosis and blepharophimosis [4, 5, 6]. To date, clinical data from 50 patients have been reported in the literature [3, 4, 5, 6, 7, 8, 9, 10, 11, 12]. Most individuals have been described as having mild to moderate ID, although detailed information on their cognitive profiles remains limited [4, 5, 6, 7, 8, 9, 10, 11, 12]. Additional features may include microcephaly or borderline small head size, hypotonia and seizures. Behavioural abnormalities and anomalies of the brain, hands and feet have also been reported [3, 4, 5, 6, 7, 8, 9, 10, 11, 12].

Here we report the clinical details of a French cohort of 29 IDDDFP patients with confirmed *BRPF1* pathogenic variants. To better characterise the phenotype and molecular spectrum, we compared our findings with data from the literature. In addition, we describe nine of these patients who underwent psychometric testing to assess the range of intellectual functioning and to delineate the cognitive phenotype.

## 2 Materials and Methods

### 2.1 Patients

Patients were referred from eight clinical genetics services in France (Besançon, Dijon, Lille, Paris [Necker‐Enfants Malades and Robert Debré hospitals] and Toulouse), with valuable assistance from the Association Francophone de Génétique Clinique. All patients were examined by physicians with expertise in clinical dysmorphology and syndromology. Blood samples were collected after informed consent was obtained from the patients or their legal representatives. This study, including all procedures, complied with the Declaration of Helsinki and French laws and regulations. Consent was also obtained for the use of photographs.

### 2.2 Molecular Analyses

DNA was extracted from peripheral blood leukocytes of patients and, where possible, their parents, using standard methods. In 17 patients, genotyping was conducted by exome sequencing (ES) or genome sequencing (GS) (single or trio) using routine techniques. Segregation of candidate variants was performed by Sanger sequencing in 8 patients. Five variants were identified using a targeted panel of 485 ID genes by standard NGS methods. Two variants were detected by array CGH, with segregation analysis performed by qPCR in 4 related patients.

Variant prioritisation was based on mode of transmission (*de novo*, autosomal recessive and X‐linked) and frequency of variants in the gnomAD database. Pathogenic *BRPF1* variants were classified according to the American College of Medical Genetics (ACMG) guidelines [13]. No other pathogenic variants were identified in ClinVar or HGMD, nor were any loss‐of‐function variants found that could explain the patients' phenotypes. All variants were confirmed by Sanger sequencing.

An analysis of the conservation of selected residues involved in missense variants was performed by multiple sequence alignment using ClustalW, including the UniProt entries P55201 ( *H. sapiens* ), K7V1M7 ( *P. troglodytes* ), D4A411 ( *R. norvegicus* ), B2RRD7 ( *M. musculus* ), and Q803M2 ( *D. rerio* ) [14]. *In silico* prediction of splice site effects were performed with SpliceAI [15, 16].

### 2.3 Neuropsychological Assessment

Neuropsychological test results were collected by the referring physician at the time of recruitment for patients P9, P12, P13, P14, P15, P16, P17, and P22. Each patient had previously been assessed by a clinical psychologist for cognitive, behavioural and/or adaptive functioning. All assessments were performed by the clinician during the session. The different scales used are summarised in Table S1 (except for patient P22, whose WISC‐V test was uninterpretable). Cognitive ability was assessed either by psychometric tests or by a developmental scale, depending on the age and ability of the patient at the time of testing. Specifically, the Wechsler Adult Intelligence Scale—III (WAIS‐III) was used for patients P14, P16 and P17; the Wechsler Preschool & Primary Scale of Intelligence—Fourth Edition (WPPSI‐IV) for patients P9, P12 and P15; and the Wechsler Intelligence Scale for Children—Fifth Edition (WISC‐V) for patients P13 and P22. The norm was defined as the interval of two standard deviations around the mean of the scores obtained by the reference population, taking into account inter‐individual variation and indicating expected functioning in the domain assessed. Each patient's results were compared with normative data for their age at the time of assessment.

## 3 Results

### 3.1 Molecular Results

**FIGURE 1:**
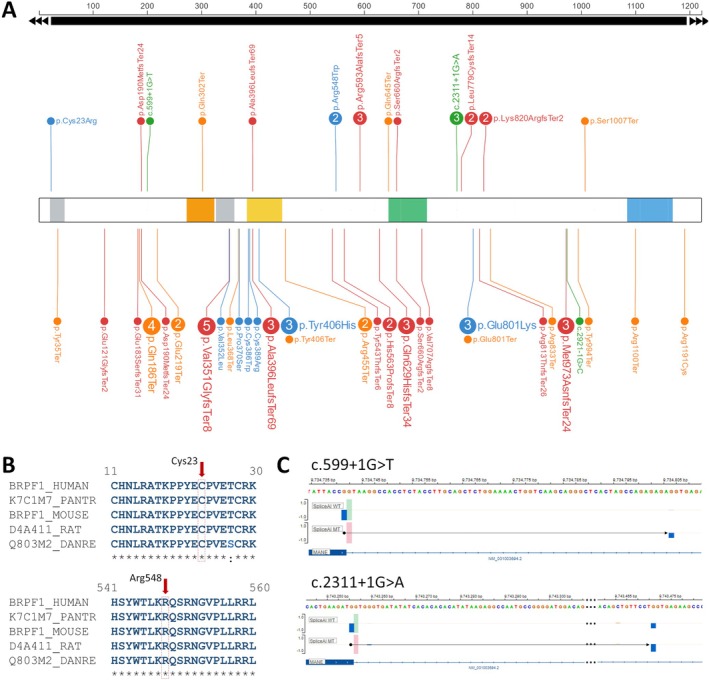
Molecular description and mapping of *BRPF1* pathogenic variants. (A) Localisation of newly reported *BRPF1* pathogenic variants (top) and previously described variants (bottom) using the online tool ProteinPaint. The different variant classes are represented by red dots for frameshift variants, orange dots for nonsense variants, blue dots for missense variants and green dots for splicing variants. The number of patients sharing the same variant, for those with a recurrence greater than 2, is indicated inside the dot. Structural domains are indicated: C2H2 zinc fingers (grey boxes), PHD‐1 domain (orange box), PHD‐2 domain (yellow box), bromo domain (green box) and PWWP domain (blue box); (B) Multiple sequence alignments of residues 11–30 and 541–560, with red arrows highlighting the positions of the missense variants at Cys23 and Arg548; (C) Predicted effect of splice variants c.599 + 1G > T and c.2311 + 1G > A using SpliceAI. Displays show predictions for donor loss with potential donor gains in distant intronic regions (64 and 217 bp, respectively).

We identified 17 unique pathogenic variants in the *BRPF1* gene in all 29 patients from 20 families (Figure 1A). Of these, 15 were point mutations, 12 of which are newly described. The frameshift mutations p.(Asp190MetfsTer14) in patient P6, p.(Ala396LeufsTer69) in patient P9 and p.(Ser660ArgfsTer2) in patient P20 have been previously reported in patients with IDDDFP [4, 17]. Two different missense variants were identified: p.(Cys23Arg) in patient P10 and p.(Arg548Trp) in patients P2 and P3. Both missense variants affect highly conserved residues (Figure 1B), were not present in the gnomAD database and were not predicted to generate cryptic splice sites [15, 18]. Splice site variants were observed in patient P4 (c.599 + 1G > T) and in a family with patients P12, P13 and P14 (c.2311 + 1G > A). These variants are not listed in the gnomAD database. The c.599 + 1G > T variant was predicted to disrupt the canonical donor splice site of exon 2 and to activate a cryptic donor splice site within intron 2 (new candidate GT at chr3:9,734,805‐9,734,806). This activation is expected to result in the retention of 64 bp of intron and the introduction of a premature stop codon, p.(Arg200Ter) (Figure 1C). The c.2311 + 1G > A variant was predicted to disrupt the canonical donor splice site of exon 7 and to activate a cryptic donor splice site within intron 7 (new candidate GT at chr3:9,743,472‐9,743,473). This activation is expected to result in the retention of 217 bp of intron and the introduction of a premature stop codon, p.(Gly771Alafs2Ter) (Figure 1C). Thirteen of the 17 unique variants were truncating variants leading to haploinsufficiency, including frameshift variants (*n* = 7), nonsense variants (*n* = 4) and complete gene deletions (*n* = 2). There was no hotspot for truncating variants (Figure 1A).

**FIGURE 2:**
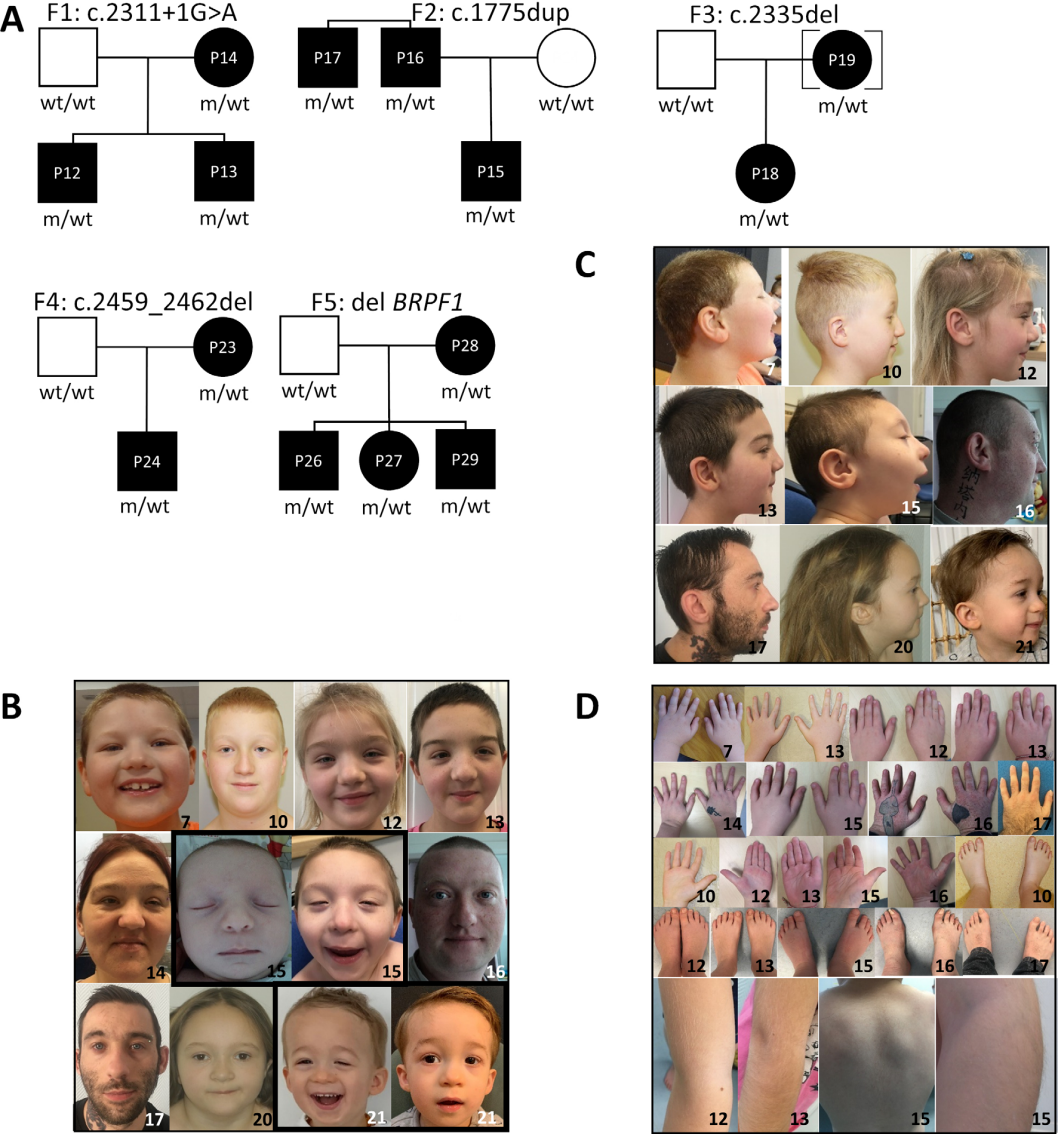
Pedigrees and clinical features of patients with pathogenic *BRPF1* variants. (A) Pedigrees of five families showing inheritance of pathogenic *BRPF1* variants; (B, C) Facial features of 10 patients; (D) Illustrations of hand and foot anomalies and cases of hypertrichosis.

Familial segregation analysis was performed in all patients except one (P4) due to lack of parental samples and was incomplete in four patients due to lack of paternal samples (P6, P10, P18 and P22). Five cases of two‐generation transmission of pathogenic *BRPF1* variants were observed in five unrelated families (Figure 2A). The first family consisted of a daughter (P12), a son (P13) and their mother (P14), all carrying the c.2311 + 1G > A splice variant. The second family consisted of a son (P15) and his father (P16), both carrying the c.1775dup variant. The third family consisted of a daughter (P18) and her mother (P19) carrying the c.2335del variant. The fourth family consisted of a son (P23) and his mother (P24), both carrying the c.2459_2462del variant. The fifth family had two sons (P26 and P29) and one daughter (P27), all with a whole gene deletion inherited from their mother (P28). All other variants were *de novo*.

### 3.2 Neurodevelopmental Features

ID/DD was observed in the majority of patients. Of the 29 patients with available data, 16 (55%) presented with ID of varying severity: six patients had mild ID, while 10 patients had moderate ID. Delayed speech and language development were noted in 13 patients (46%). Hypotonia was observed in 11 patients (40%), including one with neonatal hypotonia. Most patients had motor delay (52%). Epilepsy was documented in 4 patients (14%) and 18 patients (62%) had behavioural disorders, including attention deficit/hyperactivity (33%), low frustration tolerance (29%), inappropriate laughter (14%), anxiety (21%), agitation (15%) and autistic behaviour (15%). Sleep disturbance was reported by nine patients (31%). Brain MRI was available for 17 patients, of whom two (12%) had agenesis of the corpus callosum and one had multifocal hyperintensities in the white matter.

Neuropsychological test results were available for eight patients (Table S1). Patients P13, P16 and P17 showed heterogeneous cognitive profiles with notable discrepancies between verbal and visual scores. In patients P16 and P17, logical reasoning and conceptual abstraction were effective with visual/spatial support, but deficient with verbal input. In addition, memory capacity was impaired, although processing speed remained efficient. Patient P13 showed preserved abilities in visuoperceptive and visuoconstructive processing, while patient P9 had a strong verbal comprehension index. Intellectual disability was observed in patient P9 (mild), P13 (mild), P14 (mild), patient P15 (moderate) and patient P17 (mild). Patient P15 also showed limited attention and poor cognitive flexibility. Patient P12 had borderline intellectual functioning, with particular strength in “matrices”, reflecting efficient visual reasoning. Data from patient P22 were uninterpretable due to a highly heterogeneous cognitive profile.

### 3.3 Craniofacial Characteristics

Several common facial features were observed in the patients. Common features associated with IDDDFP included blepharophimosis (10/29; 34%), hypertelorism (14/29; 48%), ptosis (20/29; 69%), and a round face (17/27; 63%) (Table 1 and Figure 2B,C). Other notable features included up slanting palpebral fissures (7/29; 24%), epicanthus (12/29; 41%, with epicanthus inversus in 5/29), narrow palpebral fissures (10/29; 34%), palpebral oedema (8/29; 28%), low columella (9/29; 31%), bulbous nose (14/28; 50%), high palate (14/29; 48%), and retrognathia (12/29; 41%). With the exception of two patients with congenital microcephaly, microcephaly was not observed in the cohort. Ophthalmological abnormalities were found in 19 patients (66%), with strabismus present in 13 patients (48%). Refractive disorders were found in seven patients (24%), including hypermetropia (*n* = 2) and myopia (*n* = 5) (Figure 2B,C).

**TABLE 1:** Features of patients with *BRPF1* pathogenic variants.

Patient number (P) family number (F)	1	2	3	4	5	6	7	8	9	10	11	12 (F1)	13 (F1)	14 (F1)
Genomic or cDNA coordinate (NM_001003694.2)	c.904C > T	c.1642C > T	c.1642C > T	c.599 + 1G > T	*BRPF1* deletion	c.567del	c.3020_3021del	NC_000003.11:g.9646127_9808587del	c.1182_1183del	c.67 T > C	c.67 T > C	c.2311 + 1G > A		
Protein coordinate	p.(Gln302Ter)	p.(Arg548Trp)	p.(Arg548Trp)	p.(?)	N/A	p.(Asp190MetfsTer24)	p.(Ser1007Ter)	N/A	p.(Ala396Leufs*69)	p.(Cys23Arg)	p.(Cys23Arg)	p.(?)		
Inheritance	*de novo*	*de novo*	*de novo*	Adopted	*de novo*	Maternal non‐inherited, non‐tested father	*de novo*	*de novo*	*de novo*	Maternal non‐inherited, non‐tested father	*de novo*	Maternal inherited		N/A
Gender	F	M	M	M	M	M	M	M	M	M	M	F	M	F
Round face	N	N	N	N	Y	Y	N	N	N	Y	N	Y	Y	Y
Epicanthus inversus	N	Y	Y	N	N	N	N	N	N	N	N	N	N	N
Epicanthus	N	N	N	N	N	Y	N	N	Y	N	N	Y	N	N
Ptosis	Y	N	N	N	N	Y	Y	N	Y	N	N	Y	Y	N
Blepharophimosis	Y	N	N	N	N	Y	Y	N	N	Y	N	Y	Y	N
Narrow palpebral fissure	N	N	N	Y	N	Y	N	N	N	Y	N	N	N	N
Upslanted palpebral fissure	N	N	N	N	N	Y	N	N	N	N	Y	Y	N	N
Laterally extended eyebrow	N	N	N	N	N	N/A	N	N	N	N	N	Y	Y	N
Palpebral edema	Y	N	N	N	N	Y	N	N	N	N	N	Y	N	N
Hypertelorism	Y	Y	N/A	Y	Y	Y	N	N	Y	N	N	Y	Y	N
Synophrys	N	N	N	Y	N	Y	N	N	N	N	N	Y	Y	N
Strabismus	N	Y	N/A	Y	Y	Y	Y	Y	Y	N	N	N	N	N
Abnormality of refraction	N	N	N	N	N	N	N	N	N	N	N	N	N	N
Myopia	N	N	N	N	N	N	N	N	N	Y	N	N	Y	N
Low hanging columella	Y	N	N	Y	N	N	N	N	N	N	N	Y	Y	N
Bulbous nose	N	Y	N	N	N	Y	N	N	Y	N	N	Y	Y	N
High palate	Y	Y	Y	Y	N	N	N	N	Y	N	N	Y	Y	Y
Retrognathia	N	N	N	N	Y	N	N	N	Y	N	Y	N	N	N
Global developmental delay	N	Y	Y	Y	Y	Y	Y	N	Y	Y	N	Y	Y	N
Delayed speech	Y	Y	N	Y	Y	N	N	N	Y	Y	N	N	Y	N
Intellectual disability	N	Moderate	Moderate	Moderate	N/A	Moderate	Mild	N	N	Moderate	N	Milde	Moderate	Moderate
Motor delay	N	Y	Y	Y	Y	Y	Y		Y	Y	N	N	Y	N
Hypotonia	N	Y	N	N	Y	Y	N	Y	Y	N	Y	N	Y	N
Behavior troubles	Low frustration tolerance	Impulsive behavior, low frustration tolerance	Impulsive behavior	N	N	Impulsive behavior, agitation, ADHD, inappropriate laughter	N	ADHD, autistic behavior, low frustration tolerance	Anxiety	Impulsive behavior, agitation, ADHD, low frustration tolerance	N	ADHD, inappropriate laughter	Impulsive behavior, ADHD, inappropriate laughter, low frustration tolerance	N
Seizures	Y	N	N	Y	N	N	Y	N	Y	N	N	N	N	N
Brain MRI	N/A	N/A	N/A	N	Multifocal hyperintensity of cerebral white matter	N	N	N	N	N	N	N	N	N
Hypertrichosis	N	N	N	N	Y	N	N	N	N	N	N	Y	Y	N
Various hair anomalities	Y	Y	N	N	Y	Y	Y	N	N	N	N	Y	Y	N
Sleep disturbance	N	N	N	N	N	Y	N	Y		Y		Y	N	N
Gastroesophageal reflux	Y	N	N	Y	N	Y	N	Y	Y	N	Y	N	N	N
Cryptorchidism	N	N	N	N	N	N	Y	N	N	Y	N	N	Y	N
Short stature	N	N	N	N	N	N	N	N	N	N	N	N	N	N
Prominent fingertip pads	N	Y	Y		Y	N	N	N	N	N	N	N	N	N
Clinodactyly of the 5th finger	Y	N	Y	N	N	Y	Y	N	N	N	N	N	Y	N
Distal joint laxity	N	N	N	N	N	N	Y	N	Y	N	N	Y	Y	N
Hematological abnormalities	N	N	N	N	Anemia	N	N	N	N	N	N	N	N	N
Other features	Hypermetro‑pia	Bulimia			Laryngomalacia		Obesity	Facial asymmetry, plagiocephaly, recurrent viral infections	Feeding difficulties in infancy, constipation, talipes equinovarus, recurrent viral infections	Obesity, constipation	Stridor	Broad hallux	Small hands, broad hallux	

Patient number (P) family number (F)	15 (F2)	16 (F2)	17 (F2)	18 (F3)	19 (F3)	20	21	22	23 (F4)	24 (F4)	25	26 (F5)	27 (F5)	28 (F5)	29 (F5)
Genomic or cDNA coordinate (NM_001003694.2)	c.1775dup			c.2335del		c.1978_1981del	c.751C > T	c.1158dup	c.2459_2462del		c.1933C > T	NC_000003.11:g.9724807_9832101del			
Protein coordinate	p.(Arg593AlafsTer5)			p.(Leu779CysfsTer14)		p.(Ser660ArgfsTer2)	p.(Arg251Ter)	p.(Tyr387LeufsTer79)	p.(Lys820ArgfsTer2)		p.(Gln645Ter)	N/A			
Inheritance	Paternal inherited			Mother inherited	Adopted	*de novo*	*De novo*	Maternal non‐inherited, non‐tested father	Mother inherited	N/A	*de novo*	Mother inherited		N/A	Mother inherited
Gender	M	M	M	F	F	F	M	F	M	F	F	M	F	F	M
Round face	Y	Y	Y	Y	N	Y	Y	Y	Y	N	Y	Y	Y	N/A	N/A
Epicanthus inversus	Y	N	N	N	N	N	N	N	N	N	Y	N	N	N	Y
Epicanthus	N	Y	Y	N	N	Y	N	Y	N	N	N	N	N	N	Y
Ptosis	Y	Y	Y	Y	N	N	Y	Y	Y	Y	Y	Y	Y	N	Y
Blepharophimosis	Y	N	N	Y	Y	N/A	N	Y	N	N	N	N/A	N/A	N/A	N/A
Narrow palpebral fissure	Y	N	N	Y	N	Y	N	Y	Y	Y	Y	N/A	N/A	N/A	N/A
Upslanted palpebral fissure	Y	Y	N	Y	N	N	N	Y	N	N	N	N/A	N/A	N/A	N/A
Laterally extended eyebrow	Y	Y	N	N	N	Y	N	N	N	N	Y	Y	N	N	Y
Palpebral edema	Y	Y	N	N	N	N/A	N	N	N	N	N	Y	Y	N	Y
Hypertelorism	Y	N	N	Y	N	Y	Y	Y	Y	N	N	N/A	N/A	N/A	N/A
Synophrys	Y	Y	N	N	N	Y	N	Y	N	N	N	Y	N/A	N	Y
Strabismus	N	N	N	Y	N	Y	N	Y	N	Y	Y	N	N	Y	N
Abnormality of refraction	N	N	N	N	N	N	Y	N	N/A	N	Y	N/A	N/A	N/A	N/A
Myopia	Y	N	N	Y	N	N	N	N	N/A	N	Y	N/A	N/A	N/A	N/A
Low hanging columella	Y	Y	N	Y	N	Y	N	N	N	N	Y	N/A	N/A	N/A	N/A
Bulbous nose	Y	Y	N	Y	N	Y	N	N	Y	N	N/A	Y	Y	Y	Y
High palate	Y	Y	N	N	N	N	Y	N	N	N/A	N/A	Y	Y	N/A	Y
Retrognathia	Y	Y	N	Y	N	N	Y	N	Y	N	Y	Y	Y	N	Y
Global developmental delay	Y	N	N	Y	N	N	Y	Y	N/A	N	N/A	N/A	N/A	N/A	N/A
Delayed speech	N	N	N	Y	N	N	Y	Y	N/A	N	Y	N/A	N/A	N/A	N/A
Intellectual disability	Moderate	Mild	N	Moderate	Moderate	N	N/A	Mild	N/A	N	N	Mild	N/A	N/A	N/A
Motor delay	Y	N	N	N	N	Y	Y	Y	Y	Y	Y	N/A	N/A	N/A	N/A
Hypotonia	Y	N	N	Y	N	Y	Y	N	N	N/A	N	N/A	N/A	N/A	N/A
Behaviour troubles	Impulsive behavior, ADHD, inappropriate laughter, low frustration tolerance	ADHD	N	N	N	ADHD, anxiety	Agitation, low frustration tolerance	Autistic behaviour, anxiety	Agitation, low frustration tolerance	N	ADHD, autistic behaviour, anxiety	N/A	Anxiety	N/A	Anxiety, inappropriate laughter
Seizures	N	N	N	N	N	N	N	N	N	N	N	N	N	N	N
Brain MRI	Corpus callosum abormality	N/A	N/A	N	N/A	N	N	N	Corpus callosum abormality	N/A	N/A	N	N/A	N/A	N/A
Hypertrichosis	Y	Y	N	N	N	Y	N	Y	N	N	Y	N/A	N/A	N/A	N/A
Various hair anomalities	Y	Y	N	N	N	Y	Y	Y	Y	N	N/A	N/A	N/A	N/A	N/A
Sleep disturbance	Y	Y	N	N	N	N	Y	Y	N	N	Y	N/A	N/A	N/A	N/A
Gastroesophageal reflux	Y	N	N	N	N	N	Y	N	N	N	Y	N/A	N/A	N/A	N/A
Cryptorchidism	Y	N	N	N	N	N	N	N/A	Y	N/A	N/A	N/A	N/A	N/A	N/A
Short stature	N	N	N	Y	Y		Y	N	Y	Yes	Y	N/A	N/A	N/A	N/A
Prominent fingertip pads	Y	Y	N/A	Y	N/A	N/A	N	N	N	N/A	N/A	N/A	N/A	N/A	N/A
Clinodactyly of the 5th finger	Y	N	N	N	N	Y	N	Y	N	N/A	N/A	N/A	N/A	N/A	N/A
Distal joint laxity	Y	N	N	N	N	N	N	Y	N	N/A	N/A	N/A	N/A	N/A	N/A
Hematological abnormalities	N	N	N	N	Thrombocytopenia	N	N	N	N	N	N	N/A	N/A	N/A	N/A
Other features	Facial asymmetry, amblyopy, small hands, broad hallux, recurrent viral infections, atrial septal defect	Broad hallux		Amblyopy, small hands, broad hallux, scoliosis		Facial asymmetry, obesity, small hands	Hypermetropy, stridor, feeding difficulties in infancy, broad hallux	Constipation, scoliosis	Plagiocephaly, microcephaly, Feeding difficulties in infancy, recurrent viral infections	Horizontal nystagmus	Horizontal nystagmus, amblyopy, feeding difficulties in infancy, bulimia, constipation, small hands				

Abbreviations: ADHD, Attention Deficit Hyperactivity Disorder; N, absent sign; N/A, not available information; Y, observed sign.

### 3.4 Other Signs

Cutaneous abnormalities were observed in approximately one third of the cohort, including laterally extended eyebrows (8/29, 28%), hypertrichosis, mostly on the back and arms (9/29; 31%), synophrys (10/28; 36%), and various hair abnormalities (12/29; 41%), such as fine hair, sparse hair, low posterior hairline, and high anterior hairline. No abnormalities were noted in the extremities, except for clinodactyly of the fifth digit in eight patients (8/27; 30%), prominent fingertip pads in six patients (6/27; 22%), and small hands with a wide hallux in four patients (4/23; 17%) (Figure 2D).

We did not observe an increased susceptibility to ear disorders such as otitis media or deafness, but laryngomalacia was present in 2 patients (8%) and stridor in 1 patient (4%). None of our participants had congenital hearing impairment, although some had sensitivity to sound (2/25, 8%). Recurrent viral infections were seen in only four patients (14%). One patient had anaemia, and another had thrombocytopenia (both 5%). Cryptorchidism was seen in five patients (22%). Gastro‐oesophageal reflux was noted in several patients (36%), with mild feeding difficulties in infancy affecting (3/26; 12%). A few patients had constipation (14%) and bulimia (14%). Weight changes were generally normal across age groups, with only four patients being obese (14%) and no reports of decreased body weight. Short stature was noted in three patients (6/29; 21%). None of the patients were tested for growth hormone secretion.

### 3.5 Familial Variability

We found two‐generation transmissions in five unrelated families. The first familial case (F1) concerns a daughter (P12), a son (P13) and their mother (P14). The mother presented with similar morphology as their children but neuropsychological assessment shown moderate ID in the daugther whereas the son and the mother had less severe cognitive impairment (heterogenous, mild). The second familial case (F2) concerns a son (P15), his father (P16) and their cousin (P17). The son has a more severe phenotype than the two adults, both morphologically and neuropsychologically. The third familial case (F3) concerns a daughter (P18) and her mother (P19). Unlike her daughter, apart from moderate intellectual disability and blepharophimosis, the mother exhibits few clinical features. The fourth familial case (F4) concerns a daughter (P18) and her mother (P19). Both have few clinical signs and no intellectual disability. Unlike her daughter, apart from intellectual disability and blepharophimosis, the mother has no other clinical signs. The fifth family case (F5) concerns a mother (P28) and her three children (P26, P27, P29). We have little information on this family, but the mother seems less symptomatic than her children (Table 1).

## 4 Discussion

Here we present a comprehensive study of 29 new patients with IDDDFP syndrome caused by *BRPF1* pathogenic variants, expanding on the findings of over 50 previously published cases (Table 2). BRPF1, a scaffolding protein, forms part of a tetrameric complex with KAT6A, KAT6B and HAT proteins [19]. Pathogenic variants in *BRPF1* are associated with a specific neurodevelopmental disorder, Intellectual Developmental Disorder with Dysmorphic Facies and Ptosis (IDDDFP). Pathogenic variants in *KAT6A* and *KAT6B* are associated with other neurodevelopmental disorders, including Arboleda‐Tham syndrome caused by *KAT6A* variants and Say‐Barber‐Biesecker‐Young‐Simpson syndrome caused by *KAT6B* variants [20, 21]. We identified pathogenic *BRPF1* variants distributed across all domains of the BRPF1 protein. Our results, in agreement with other studies, do not show a clear genotype–phenotype correlation [9].

**TABLE 2:** Comparison of newly described patients and previously published *BRPF1* variants.

Features	Series	Previous publication
	[Total (percentage%)]	[Total (percentage%)]
Neurological manifestations		
Intellectual disability	16/29 (55%)	35/49 (71%)
Delayed speech and language development	13/28 (46%)	41/50 (82%)
Intellectual disability, mild	6/16	N/A
Intellectual disability, moderate	10/16	N/A
Motor delay	15/29 (52%)	39/49 (80%)
Hypotonia	11/29 (40%)	29/59 (49%)
Seizure	4/29 (14%)	7/59 (12%)
Low frustration tolerance	8/28 (29%)	N/A
Abnormal aggressive, impulsive or violent behavior	6/27 (22%)	N/A
Agitation	4/27 (15%)	N/A
Attention deficit hyperactivity disorder	9/27 (33%)	10/52 (19%)
Autistic behavior	4/26 (15%)	4/52 (8%)
Anxiety	6/29 (21%)	1 (N/A)
Inappropriate laughter	4/25 (14%)	N/A
Sound sensitivity	2/29 (7%)	N/A
Ophtalmological features		
Laterally extended eyebrow	8/29 (28%)	N/A
Narrow palpebral fissure	10/29 (34%)	N/A
Upslanted palpebral fissure	7/29 (24%)	4 (N/A)
Blepharophimosis	10/29 (34%)	17 (N/A)
Ptosis	20/29 (69%)	26 (N/A)
Palpebral edema	8/29 (28%)	N/A
Strabismus	13/27 (48%)	6/15 (40%)
Myopia	5/29 (17%)	N/A
Hypermetropia	2/29 (7%)	6 (N/A)
Abnormality of refraction	7/29 (24%)	41/53 (77%)
Horizontal nystagmus	2/29 (7%)	1 (N/A)
Amblyopia	3/29 (10%)	2 (N/A)
Other craniofacial features		
Round face	17/27 (63%)	8 (N/A)
Facial asymmetry	3/28 (11%)	3 (N/A)
Plagiocephaly	2/28 (7%)	N/A
Microcephaly	2/29 (7%)	14/52 (27%)
Epicanthus inversus	5/29 (17%)	N/A
Epicanthus	12/29 (41%)	7 (N/A)
Hypertelorism	14/29 (48%)	13 (N/A)
Synophrys	10/28 (36%)	N/A
Low hanging columella	9/29 (31%)	N/A
Bulbous nose	14/28 (50%)	6 (N/A)
High palate	14/29 (48%)	N/A
Retrognathia	12/29 (41%)	N/A
Other signs		
Stridor	1/28 (4%)	2/15 (13%)
Laryngomalacia	2/28 (7%)	5/15 (33%)
Hypertrichosis	9/29 (31%)	N/A
Various hair abnormalities	12/29 (41%)	5 (N/A)
Obesity	4/28 (14%)	2 (N/A)
Sleep disturbance	9/29 (31%)	N/A
Gastroesophageal reflux	9/29 (31%)	4/15 (27%)
Feeding difficulties in infancy	3/26 (12%)	24/42 (57%)
Bulimia	4/29 (14%)	N/A
Constipation	4/29 (14%)	N/A
Cryptorchidism	5/27 (19%)	N/A
Short stature	6/29 (21%)	17/42 (40%)
Small hand	4/27 (15%)	5/25 (20%)
Prominent fingertip pads	6/27 (22%)	N/A
Broad hallux	4/27 (15%)	N/A
Clinodactyly of the 5th finger	8/27 (30%)	10/25 (43%)
Talipes equinovarus	1/28 (4%)	3 (N/A)
Distal joint laxity	7/27 (26%)	5 (N/A)
Scoliosis	2/27 (7%)	N/A
Atrial septal defect	1/25 (4%)	9/49 (18%)
Recurrent viral infections	4/28 (14%)	N/A
BRAIN MRI findings		
Anomaly of Corpus callosum	2/17 (12%)	2/15 (13%)
Cerebral malformation	1/17 (6%)	13/26 (50%)

Abbreviations: MRI, Magnetic Resonance Imaging; N/A, not available information.

A range of communication impairments have been described in *BRPF1*‐related disorders, including phonological impairments, altered nasality, childhood apraxia of speech, and expressive and receptive language disorders [22]. Despite the high incidence of communication impairments, the patients tend to have a comparatively stronger cognitive profile than those with other neurogenetic chromatin‐related conditions associated with intellectual disability [23]. Thus, intellectual disability is not always present in *BRPF1*‐related disorders, but 71% (35/49) of previously reported cases had cognitive abilities ranging from borderline to severe, which is consistent with the neurodevelopmental features observed in our cohort (55%) [5, 9, 11, 12, 22]. In our study, we did not find severe impairment, but rather mild (21%) to moderate (34%) difficulties. Studies in mice have shown that *BRPF1* haploinsufficiency leads to deficits in learning, memory and behaviour [24, 25]. Consistent with previously reported *BRPF1* patients, some individuals in our series presented with autistic behaviours without formal autism assessment (4/26) [4, 22]. They may also present with other behavioural disorders, including hyperactivity (9/27), low frustration tolerance (8/29), agitation (4/27), aggressive or impulsive behaviour (6/27), anxiety (6/29), inappropriate laughter (4/25) and bulimia (4/29), despite high levels of adaptive behavioural functioning [22]. These findings suggest that autism or autism spectrum disorders may be underdiagnosed and warrant more thorough assessment in individuals with *BRPF1*‐related disorders. Similarly, sleep disturbances appear to be relatively common in our cohort, although they have not been widely reported in the literature. Hypotonia is a characteristic feature of the syndrome, as observed in our cohort (11/29) and well documented in the literature (29/59). Epilepsy, although more common in our cohort (4/29) than in the literature (7/59), remains a relatively rare and non‐specific manifestation.

Among the five families reported here, phenotypic differences were observed between family members carrying the same pathogenic variant, affecting intellectual ability, dysmorphic features and malformations, suggesting an intrafamilial variability. To date, three multiplex *BRPF1* families have been described, and some patients carry a pathogenic *BRPF1* variant inherited from an asymptomatic or mildly symptomatic parent without intellectual disability [4, 7, 26]. Our series further demonstrates that the clinical expression is variable, with non‐homogeneous clinical signs observed between patients, even within the same family. The pathogenic variants in our five families were not inherited from healthy parents, suggesting a relatively high penetrance. The genetic profile of *BRPF1*‐related disorders appears to be characterised by variability in intra‐ and extrafamilial clinical expression, probably influenced by genetic background and environmental factors.

The clinical features of patients with *BRPF1* pathogenic variants show considerable variability. Common physical features include ptosis, blepharophimosis, hypertelorism, epicanthus, round face and a broad nasal bridge or bulbous nose [4, 5, 7, 21]. Notably, a high palate and retrognathia are more common in our cohort compared to previously reported cases (14/29, 48% vs. 27% [22]; 12/29, 41%) [7, 9, 10, 21]. Our study also identified novel phenotypic features such as palpebral oedema (8/29, 28%), laterally extended eyebrows (8/29, 28%), low hanging columella (9/29, 31%), hypertrichosis (9/29, 31%), synophrys (10/28, 36%) and various hair abnormalities (12/29, 41%, including fine hair, sparse hair, low posterior hairline and high anterior hairline). Dental abnormalities were not observed in our patients, although more systematic investigation is warranted to confirm this finding. These newly identified clinical features may help to differentiate *BRPF1*‐related disorders from other conditions with overlapping phenotypes, such as those related to *KAT6A* and *KAT6B* [20, 21].

Loss of *BRPF1* has been shown to affect the transcriptional regulation of several key transcription factors, including Pitx2, Hmx1 and Pax6, which have been implicated in a wide range of ocular developmental abnormalities [27]. This likely accounts for the significant frequency and variability of ocular defects observed in our cohort and reported in the literature. In the literature, patients (41/53) are mainly reported to have visual impairments including strabismus, hypermetropia and myopia, with sporadic cases of coloboma, microphthalmia, nystagmus and amblyopia [6, 17, 22, 28, 29]. Our cohort had similar findings: strabismus (13/27), myopia (5/29), hypermetropia (2/29), nystagmus (2/29), amblyopia (3/29) and cataract (1/29). In addition, two patients with pathogenic frameshift variants were described with optic neuropathy consistent with syndromes involving KAT6A/KAT6B complex defects [30, 31]. The authors also reported associated developmental and speech delays, intellectual disability and dysmorphic features [11].

Cerebral malformations are commonly reported in the literature (13/26), including periventricular nodular heterotopia, Arnold‐Chiari malformation and abnormalities of the corpus callosum (e.g., fineness, hypoplasia, dysgenesis and agenesis) [4, 6, 11, 22]. In contrast, these abnormalities were rarely observed in our cohort (3/17), although the types of malformations are consistent. This discrepancy may be due to the fact that MRIs are not routinely ordered but based on clinical indications. Nevertheless, brain malformations have been associated with haploinsufficiency of the *BRPF1* gene. Pathogenic *BRPF1* variants have been shown to cause neocortical abnormalities, including partial agenesis of the corpus callosum and impairments in hippocampal granule cells and cortical pyramidal neurons in animal models [24, 27].

No growth retardation was observed in our cohort, and short stature was noted in three patients (6/29, 21%), whereas short stature is relatively common in IDDDFP patients reported in the literature (17/42, 40%). None of the patients in our cohort were tested for growth hormone secretion. In addition, some patients had limb anomalies such as clinodactyly, brachydactyly, brachymetacarpia, camptodactyly, syndactyly and cavus anomalies [4, 5, 10, 17, 22], as well as congenital talipes equinovarus [4, 10]. Similar findings were observed in our cohort, with short hands (6/29, 21%) and clinodactyly (8/27, 30%) being the most common signs.

Feeding problems were observed in 12% of our series (3/26) compared with 57% of patients reported in the literature (24/42). Gastro‐oesophageal reflux was a common problem in our series, affecting 9 of 29 patients (31%), compared with 4 of 15 participants (27%) in the report by Morison and collaborators [22]. They also described laryngomalacia in 5 participants (33%) and tracheomalacia in 2 participants (13%), both of which were less common in our series: one case of laryngomalacia (3%) and two cases of stridor (7%). Ear infections in early childhood were not more common than in other young children (1/25, 4%), contrary to previous publications [22]. None of our participants had congenital hearing impairment, although some had sensitivity to sound (2/29, 7%). Cryptorchidism, not previously reported in the literature, was relatively common in our cohort, occurring in 5 of 27 patients (19%).

Cardiac anomalies were reported as a less common clinical finding, occurring in 9 of 49 cases. Specific anomalies include patent ductus arteriosus, atrial septal defect, ventricular septal defect [5, 10, 22], and atrioventricular (AV) nodal pauses and blocks [26]. Similarly, we observed one minor heart defect in our cohort (1/25). The expression of *BRPF1* in the heart during early embryonic development may explain the occurrence of cardiac malformations. However, the effect of *BRPF1* variants on cardiac tissue has not yet been studied and further research is needed to understand the potential mechanisms involved.

A previously reported patient presented with anaemia (2/3) and thrombocytopenia (1/3) [10]. In our cohort, two different patients presented with haematopoietic abnormalities without evidence of bone marrow damage (7%, 1/25 with anaemia and 1/25 with thrombocytopenia). This suggests that these abnormalities may represent a novel aspect of the phenotype associated with *BRPF1* pathogenic variants. Selective deletion of *BRPF1* in mouse blood cells has been associated with bone marrow failure, suggesting a potentially essential role in foetal haematopoietic stem cell development and haematopoiesis [32]. However, as highlighted by Kose and collaborators, additional research, particularly through in vitro modelling studies, to elucidate the underlying mechanisms [10]. Such studies could also guide the development of standardized hematological follow‐up procedures to ensure timely detection and management of potential complications in patients with *BRPF1* pathogenic variants.

Our study, the largest cohort of *BRPF1‐*related disorders to date, advances understanding of IDDDFP but is limited by its sample size, reflecting the rarity of the condition, and variable depth of clinical data, particularly in neuropsychological and haematological assessments. Further research with larger cohorts and longitudinal studies is needed to validate findings and refine management strategies.

In conclusion, we present here the largest cohort of individuals with IDDDFP to date, including detailed descriptions of familial inheritance. Our findings confirm that *BRPF1*‐related disorders are typically associated with characteristic facial features and intellectual disability. However, we have also highlighted the considerable variability in clinical presentation, including morphological, malformative and neurodevelopmental aspects. Newly described features include specific abnormalities of the phanera. To improve patient care and further our understanding of *BRPF1*‐related disorders, we recommend that all individuals with pathogenic *BRPF1* variants undergo regular ophthalmological surveillance.

## Author Contributions

C.C. and T.S. wrote the manuscript. C.C., M.T., J.L.C., C.P., A.L.B., F.T.M.T., J.L. and T.S. performed the experiments, and the data interpretation. C.C., M.R., E.B.B., O.B.B., B.D.B., L.F., C.H., J.L.C., G.L., O.P., A.P., M.R., C.T., C.V.D., C.V. and J.G. collected and evaluated the clinical and genetic data C.C., J.G. and T.S. revised the manuscript. All authors discussed the results, commented on the manuscript, and approved the final manuscript.

## Conflicts of Interest

The authors declare no conflicts of interest.

## Supporting information


**Table S1.** Developmental and cognitive assessments.

## Data Availability

The data that support the findings of this study are available from the corresponding author upon reasonable request.
