# Supplementary material for: The Phenotypic and Genotypic Spectrum of BRPF1‐Related Disorder: 29 New Patients and Literature Review
Source: Clin Genet. 2024 Dec 29;107(5):527–40. doi: 10.1111/cge.14688 (PMC11973018; doi:10.1111/cge.14688)
Supplement: Supplementary file 1 — Table S1. Developmental and cognitive assessments. [file CGE-107-527-s001.docx]

**Supplemental Ta****ble 1. Developmental and Cognitive Assessments**

| Patients | Ages at the assessment | Tests | Conclusions | Verbal Intelligence Quotient | QIP Quotient Intellectuel Performance | Verbal Comprehension Index | Perceptual Reasoning Index | Working Memory Index | Processing Speed ​​Index | Visual Spatial Index |
| --- | --- | --- | --- | --- | --- | --- | --- | --- | --- | --- |
| P9 | 6y 5m | WPPSI-IV | mild ID |  |  | 72 | #N/A | #N/A | #N/A | 69 |
| P12 | 5y 11m | WPPSI-IV | borderline |  |  | 63 | 77 | 75 | 77 | 72 |
| P13 | 9y 11m | WISC-V | mild ID |  |  | 50 | 74 | 65 | 60 | 84 |
| P14 | 38y 2m | WAIS-III | mild ID | 53 | 66 | 65 | 68 | 50 | 97 |  |
| P15 | 4y 7m | WPPSI-IV | moderate ID |  |  | 45 | 45 | 45 | 45 | 60 |
| P16 | 31y | WAIS-III | heterogeneous cognitive profiles | 55 | 84 | 63 | 87 | 53 | 80 |  |
| P17 | 28y 5m | WAIS-III | mild | 54 | 72 | 63 | 73 | 50 | 78 |  |

*#N/A: not available information; y: Years; m: Months*
